# Performance Analysis of Advanced Feature Extraction Methods for Manufacturing Defect Detection via Vibration Sensors in CNC Milling Machines

**DOI:** 10.3390/s26072195

**Published:** 2026-04-02

**Authors:** Gürkan Bilgin

**Affiliations:** Department of Electrical-Electronics Engineering, Burdur Mehmet Akif Ersoy University, 15200 Burdur, Türkiye; gbilgin@mehmetakif.edu.tr; Tel.: +90-248-2132779

**Keywords:** CNC milling machines, empirical mode decomposition, Hilbert–Huang transform, process quality, signal feature extraction, vibration signals, time-domain analysis, frequency-domain analysis, time–frequency-domain analysis

## Abstract

This study investigates the effectiveness of various feature extraction methods applied to vibration signals for the automatic detection of production defects in CNC (Computerised Numerical Control) milling machines. A dataset consisting of real-world data collected from CNC machines equipped with accelerometers was used. The objective of the study is to compare three main groups of techniques: time-domain analysis (TDA), frequency-domain analysis (FDA), and time–frequency-domain analysis (TFA). The findings indicate that basic TDA features lack the necessary sensitivity to accurately distinguish between Good Processing (GP) and Bad Processing (BP) states. Frequency-domain methods, such as the Fast Fourier Transform (FFT), median frequency calculation, and the Welch periodogram, provide better insights but still have limitations. The most effective results are obtained with TFA methods, particularly Empirical Mode Decomposition (EMD) and the Hilbert–Huang Transform (HHT), which reveal deeper signal characteristics. Following the feature optimisation studies, it was determined that a combination of four features—FMED, IMF2, IMF5 and WPT26—yielded the optimal performance, with an accuracy of 91.48%. The incorporation of a fifth feature resulted in information saturation within the model and did not improve performance. This study makes a novel contribution to literature by conducting an in-depth investigation into the most effective feature extraction and selection techniques for achieving robust discrimination between GP and BP productions using vibration signals in CNC milling processes. Conclusively, TFA features, supported by advanced signal processing, offer a strong basis for reliable, automated defect detection in CNC milling operations.

## 1. Introduction

In industrial production, automation studies involve installing various sensors on machines to enable automatic fault detection. Especially with the development of sensors, digital controllers, communication systems and artificial intelligence algorithms, these automated systems have also improved significantly. Vibration signals from vibration sensors are often used as source signals for feature extraction in failure or product fault detection studies. Equipment that has been positioned incorrectly or has aged over time will cause vibration by creating an oscillating motion. Frequency and amplitude distortions in the signal indicate a problem with the machine’s operation [1]. The analysis of vibration signals has been proven to be an effective method of monitoring machine operating conditions and faults, with the objective of minimising failure costs [2,3,4]. Feature extraction from vibration signals plays a significant role, especially in the fault diagnosis of devices with rotary motors and in the identification of faulty products. CNC (Computerised Numerical Control) systems, which use rotary motors to machine various materials without operator intervention, are powerful, practical, and basic pieces of equipment in industrial production [5]. Even though advanced industrial production systems are quicker and more efficient, they can also be extraordinarily complex. This complex configuration has the potential to induce a multitude of complications, including device impairment and signal degradation, which can be attributed to environmental factors, accelerated manufacturing processes, and inadequate connections [6]. Such failures may cause interruptions and machine failures on the production line, as well as product defects. During the production process, machine parameters may change over time due to various environmental factors, including humidity, temperature, pressure, vibration, wear, corrosion, and long-term machine operation. Therefore, the machines require periodic maintenance. During periodic maintenance, operations such as part replacement, lubrication, and machine cleaning are performed [7]. Such operations further increase the complexity of the systems. Therefore, non-stationary and complex vibration signals contain critical information about the condition of rotating machines. Feature extraction from vibration signals is imperative for identifying potential faults in industrial equipment, with the ultimate objective of enhancing reliability and efficiency.

The selection of the sensor for acquiring vibration data may vary depending on the application and environmental conditions. Accelerometers, microelectromechanical systems (MEMS), piezoelectric sensors, gyroscopes, and inertial measurement units are frequently preferred for sensing vibrations and motion in rotating machines [8,9,10,11]. Accelerometers are a useful tool for detecting vibration signals due to their high sensitivity, ease of operation, wide frequency bandwidth, and real-time operation. Numerical processing of acceleration values and detection of minimal displacement motions are simpler with accelerometers [12,13]. Kafeel et al. investigated fault detection using physically measured vibration signals from induction motors in large industrial plants. In their study, the researchers used a 3-axis wireless accelerometer with a sampling frequency of 1kHz to obtain vibration signals, which they combined into a single signal using the composite of the axes [14]. In different studies, vibration signals of rotating machines have been obtained by using low-cost accelerometers with various designs. Chaudry et al. developed an accelerometer design that they used to detect different fault combinations in the motor [15]. In another alternative study on low-cost rotating machine health analysis, an electric bicycle wheel motor was used for the experiments. A MEMS accelerometer (MMA8451Q) was used to acquire vibration signals, and the ESP32 microcontroller was used for data evaluation. As a result, the success of the implemented wireless sensor network system was emphasised [16]. In another system developed for monitoring vibration signals in rotating structures, a wireless, simple, and inexpensive system is proposed using a Raspberry Pi and a MEMS accelerometer. The system’s accuracy is evaluated using a piezoelectric wired accelerometer as a reference. Both accelerometers are mounted on the tip of the rotating blade [11]. Ghemari et al. obtained more accurate vibration results. In their study, they proposed a new mathematical model for a piezoelectric accelerometer. A comparison of the results with the literature showed that the vibration analysis technique improved [17]. Feldman et al. used dual Off-The-Shelf MEMS accelerometers for vibration analysis of a MEMS micro-turbine supported on micro-ball bearings due to their wide bandwidth, volume, sensitivity, and cost. Experiments were carried out by mounting the accelerometers directly to the stator. It was emphasised that an accelerometer is a valuable tool for monitoring, fault detection, and characterisation of rotary microturbine systems, owing to its wide bandwidth and high sensitivity [18].

It is not easy to cope with the requirements frequently encountered in industrial production processes, such as fault detection in rotating systems, error control in production, and system status monitoring. Current research trends emphasise extracting hidden features within the harmonic components of vibration signals to address these challenges through AI-based frameworks [19,20]. Altaf et al. attempted to detect bearing failures from vibration signals. The researchers used Empirical Mode Decomposition (EMD) and Fourier analysis for feature extraction. The application of these extracted features to machine learning inputs resulted in an accuracy of 99.13%, as reported in [21]. Tnani et al. attempted to detect anomalies in machine processes using datasets. Utilising an effective two-stage learning approach, they attained an F1 score of 90.3%, thereby demonstrating the efficacy of their methodology [22]. In another study, an excellent performance was achieved with TSFRESH (version v0.11.0, Blue Yonder GmbH, Karlsruhe, Germany; a Python package used for systematic feature engineering from time series). In this application, feature extraction can be performed automatically without requiring much human expertise [23]. Tama et al. emphasised the high efficacy of vibration signal features for detecting faulty operations and faulty production processes in industrial machines in their review article. The authors also discussed the effectiveness of deep learning and machine learning methods in this field [24]. Liu et al. aimed to classify vibration signals for fault detection in rotating machines using an autoencoder-based super graph feature learning method and achieved successful results [25]. In another study, vibration and airborne sound were analysed by acquiring data with sensors. This was complemented by the determination of lateral and angular shaft alignment errors within the gear mesh. The assessment employed state indicators, such as root-mean-square energy, kurtosis, and skewness, to ensure a comprehensive analysis [26]. In a separate study on feature extraction for fault diagnosis, two main groups were evaluated: frequency-domain (FDA) and time–frequency (TFA) analysis. Root mean square (RMS), mean, variance, skewness, kurtosis, and crest factor were used as time-domain features. For both time and frequency domains, Fast Fourier transform (FFT), power spectrum, bispectrum, cyclostationarity, Short-Time Fourier Transform, Wigner Ville distribution, spectrogram, wavelet transform, and matching pursuit. He evaluated the advantages and disadvantages of these techniques relative to one another [27]. Moreover, a series of experiments was conducted at Case Western Reserve University to evaluate the condition of rolling bearings and identify failures in industrial applications. The experimental results, which used a range of feature parameters to analyse vibration signals, demonstrated an accuracy of 99.83%, as reported in another study [28].

Studies have identified fault detection methods based on feature extraction from vibration signals as a preferred approach due to several advantages, including the use of inexpensive accelerometers, high detection efficiency, and real-time vibration monitoring [29]. A general tendency has been observed in the extant literature for studies to focus on time-domain, frequency-domain, and time–frequency domain methods for feature extraction from vibration signals [30,31,32]. Li et al. analysed the performance comparison of time, frequency and time–frequency-domain features extracted from vibration signals from rotating mechanical systems. The researchers reported that optimal classification results were obtained with time–frequency-domain feature extraction, whereas time-domain features yielded the least effective outcomes [30]. In a separate study, the time–frequency-domain feature-extraction method was identified as a preferred approach for fault detection in condition-based maintenance and vibration signal analysis. The study emphasised that the fault condition can be monitored via signal analysis, and its effectiveness was demonstrated using the Daubechies wavelet (db14) in the discrete wavelet transform (DWT) [31]. In a similar vein, Kheir-Eddine et al. used vibration signals to predict wear and failure in a special-purpose milling machine. The authors concluded that Wavelet Packet Decomposition (WPD) was the most suitable method for extracting features from the accelerometer signals. Additionally, methods such as kurtosis and skewness, entropy, variance, root mean square, shape factor, and peak factor were applied to the raw signals. The study noted the superior performance of the WPD method, achieving 98% accuracy in feature extraction for blade wear detection and 98% accuracy in the classification stage of blade wear detection with Support Vector Machines (SVM). The study also reported a 95% accuracy rate with Random Forest [33]. In a related study, an accelerometer was used in conjunction with the Time Direct Analysis, power spectral density (PSD), Singular Spectrum Analysis (SSA), and Wavelet Packet Transform (WPT) methods to extract features from vibration signals, thereby enabling evaluation of surface quality. Consequently, they cited the efficacy of the SSA and WPT methodologies, though they noted that SSA entails substantial computational requirements. During the study, the authors placed particular emphasis on the WPT method, arguing that it is the most effective method for monitoring surface quality in CNC machining [34]. A study was conducted to determine the efficiency of the Complete Ensemble Empirical Mode Decomposition with Adaptive Noise and Multi-Scale Dispersion Entropy methods for identifying vibration faults in power transformers. The study concluded that both methods are effective for feature extraction [35]. Olalere and Olanrewaju applied the EMD method to the Intrinsic Mode Function (IMF) feature vector for classification, with the aim of detecting flank wear [36]. In a similar study, time analysis statistical methods such as RMS, kurtosis, skewness, peak to peak, crest factor, shape factor, impulse factor, margin factor and EMD were used for feature extraction in condition monitoring and fault detection for rolling element bearings (REBs) and mathematical analysis was presented to select the best IMFs [37]. In a study by Kumar et al., vibration signals were recorded during a shaft rotation speed of 3000 rpm in a rotor-bearing system. The signals were analysed using FFT, the wavelet transform, and EMD. It was determined that the FFT of the signals shows peaks at the rotational speed and its multiples. Furthermore, it was ascertained that the approximation coefficient A3 and the detail coefficient D4 in wavelet analysis most accurately express the operating speed. Additionally, the second-level decomposition of the Ensemble Empirical Mode Decomposition (EEMD) exhibited the maximum vibration level at the shaft operating frequency [38].

The augmentation of artificial intelligence methodologies increases the automation and mechanisation of systems. Nevertheless, the use of large databases is essential to increase the reliability and robustness of these systems’ designs. For the development of industrial systems, several databases have been developed that contain data obtained by integrating sensors into the system during the production process, as described in the literature. The NASA milling dataset is a typical example of this type of dataset. The data under consideration consists of signals from vibration, acoustic emission, and current sensors [39]. Another milling dataset, designated SMART LAB, was developed at the University of Michigan to detect vehicle corrosion and poor engagement [40].

The datasets mentioned above were acquired under laboratory conditions and are short-term recordings [39,40]. In the present study, a dataset of 2 years of long-term recordings from CNC milling machines using vibration sensors in industrial production, rather than a laboratory environment, was preferred [22]. The adoption of this approach was driven by the objective of attaining more realistic and reliable results. The use of these datasets facilitates the development of more reliable and realistic artificial intelligence models, thereby reducing losses and failures in production processes.

The present study focuses on extracting features prior to implementing artificial intelligence in a database of vibration-based signals obtained from CNC freezing machines during the production process. The main purpose of this study is to highlight some of the important aspects of feature extraction used in artificial intelligence-based systems in situations where there is a need to be able to perform faulty or faultless production classification by using data from different CNC freezing machines during different processes, and to draw attention to the main differences between feature extraction methods. In the context of such fault detection studies, preprocessing signals or images prior to artificial intelligence and feature extraction over these data constitutes a crucial step. An evaluation of the mean, RMS, standard deviation, skewness, and kurtosis was conducted to determine the time-domain statistical properties. For the frequency domain, the FFT analysis, Welch periodogram (WP) and median frequency (MF) values of the signals were initially obtained, after which the features were extracted from these values. The effects of these features on the decomposition were then analysed. Subsequent analyses in the frequency and time–frequency domains were executed systematically to ensure high-resolution feature characterisation. The WPT, EMD, and HHT were applied, and the results of the analyses were evaluated relative to one another.

## 2. Materials and Methods

This section provides detailed information about the database used and gives an overview of the study. The following sections concern preprocessing and the mathematics of feature extraction.

### 2.1. Dataset Description

**Figure 1:**
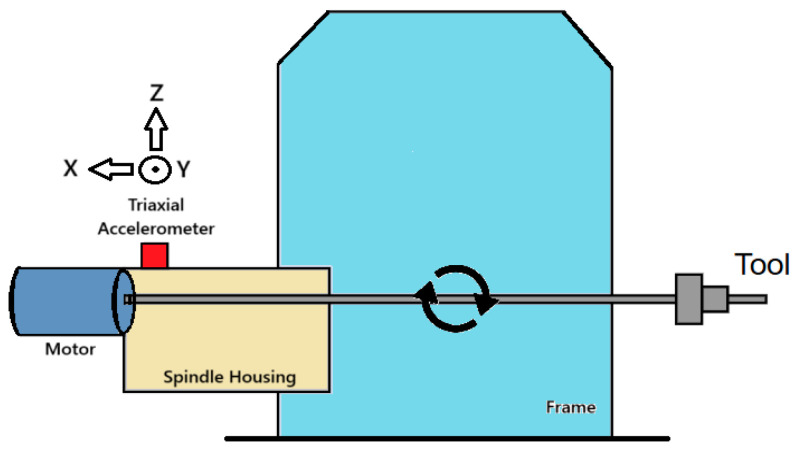
Schematic representation of the experimental setup utilised for creating the database [22].

The data used in the analyses were retrieved directly from a public dataset compiled by Tnani et al. [22]. The benchmark dataset used in this study is obtained from a real-world industrial production plant, which provides a comprehensive view of Brownfield CNC milling machine operations, capturing the complexity of monitoring precision and high-speed production across three machines (M01, M02, M03) from October 2018 to August 2021. Vibration signals were recorded using a triaxial accelerometer mounted on the rear bed on each machine as shown in Figure 1. Signals were taken at 2 kHz sampling frequency with the Bosch CISS sensor (Robert Bosch GmbH, Stuttgart, Germany) [22]. For the purposes of this study, all analyses were performed within a bandwidth of 5 Hz to 1 kHz, in accordance with the Nyquist–Shannon sampling theorem.

Since machines process various aluminium parts (such as drilling and cutting) using different tools and setups, the dataset consists of 15 segments (OP1, OP2, OP3, …, OP14) that represent different sets of operations. This database has been meticulously curated to support scientific studies in machine learning, with the primary objective of tackling multifaceted challenges in data-driven modelling and analysis. However, the dataset exhibits some limitations: features are subject to drift across distinct machines and temporal spans, it encompasses a diverse array of tool operations within the production process, and it presents an imbalance in sample distribution across classes.

For each process, the dataset includes the month and year in which the data was measured, the name of the machine and process from which the data was taken, the acceleration values measured in three axes, and the label information about the product produced after the process as “good” or “bad”.

**Figure 2:**
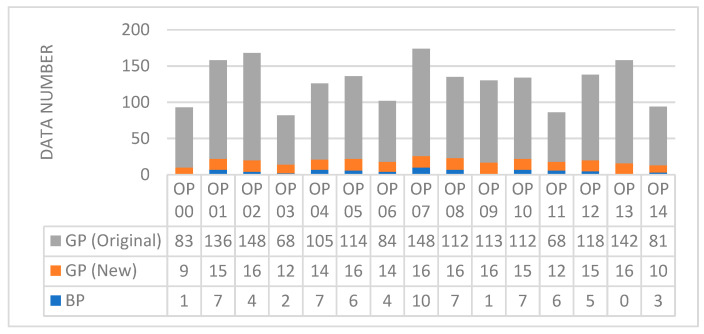
Comparison of the data distribution across various operations before and after the undersampling of Good Parts (GP) relative to Bad Parts (BP).

The dataset under consideration contains 1702 measurements. Since the motivation of this study focuses on the GP (Good Processing—“good”) and BP (Bad Processing—“bad”) distinction, other information in the dataset, such as the number of machines, the number of processes, etc., is ignored. A total of 70 of these records are from BP. Upon examination of the dataset, a significant imbalance in the number of BP and GP data was observed. Given the potential for this imbalance to compromise the study’s accuracy, all GP measurements were deemed ineligible for inclusion. However, upon a thorough examination of the database, it was observed that the number of GP data points is significantly higher than the number of BP data points. Here, the number of BP data points is 70, while the number of GP data points is 1632. Consequently, the BP/GP ratio is 0.0429 [22]. As the primary objective of the study is to distinguish between GP and BP, it is crucial for classification studies that the datasets are similar in size and that their distributions are homogeneous. For this reason, all BP data were included in the study in the same manner. For GP data, however, for the purpose of this study, only the initial measurements obtained from consecutive measurements with label “000” (such as “M01_Aug_2019_OP00_000.h5”) were considered. In addition, the wear times of the machines and operational differences were taken into account, and data were collected from all components to achieve a data distribution as homogeneous as possible. Following the undersampling process, the BP/GP ratio was increased to 0.330. In this study, 282 data points were selected from the dataset. Of these, 70 are classified as BP data, while the remaining 212 are designated as GP data. The effectiveness of feature extraction methods for discriminating GP or BP regardless of the machine operation number and operating speed on the database is compared. Currently, the evaluation of feature extraction methods is becoming increasingly significant due to the growing prevalence of the condition of CNC-produced materials as a subject of interest in the research community. Currently, the evaluation of feature extraction methods is becoming increasingly significant as automatic detection of the condition of material produced by CNC is among the popular topics [6,41,42]. The database distribution, taking into account the original dataset, is shown in Figure 2.

When the data in Figure 2 is examined, a new dataset has been created by considering all the dates on which records were taken for each machine and each operation. In Figure 2, GP (original) refers to the number of GP data in the public database, while GP (New) indicates the number of GP data in the dataset created for this study. BP data counts have been taken directly from the original dataset.

Consequently, both BP and GP measurements were obtained from each machine’s data; data samples were collected for each operation, and at least one sample was collected for each date. Therefore, this approach has ensured a more balanced representation across the dataset, significantly reducing any potential impact on the overall balance of the study. Thus, the class imbalance is adjusted to a level that ensures a reliable analysis. Anomalies BP in real-world data are extremely rare. To prevent the GP class from dominating the analysis, undersampling has been applied to the GP data, reducing the dataset’s severe imbalance to reasonable levels. Furthermore, the undersampling process will not affect temporal or machine drifts.

### 2.2. Overview of the Study

**Figure 3:**
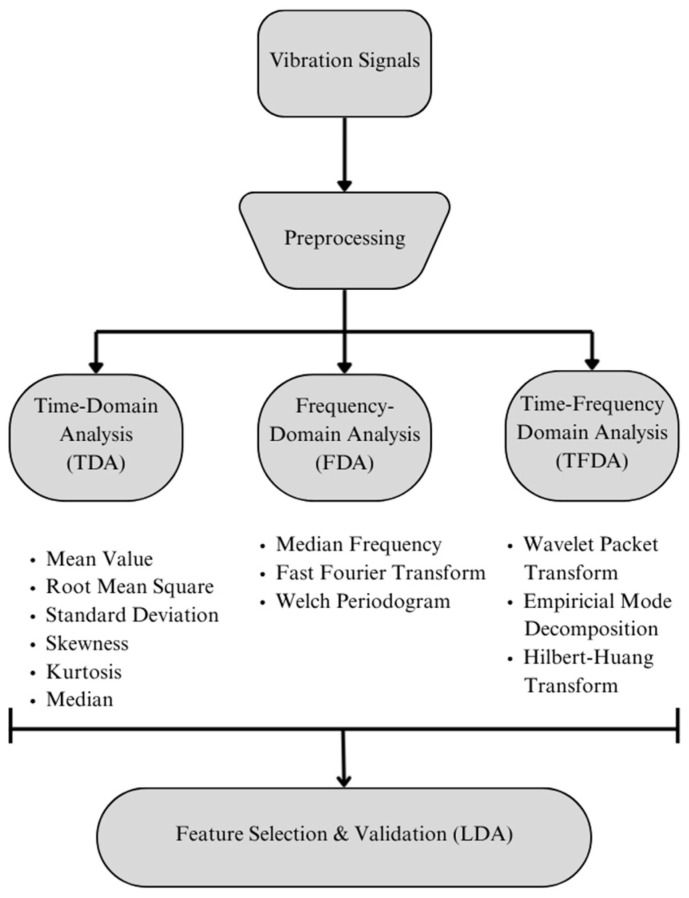
Main block diagram of the study.

The utilisation of vibration signals, transmitted to the digital environment via sensors affixed to various industrial machinery, necessitates the examination and interpretation of signal changes. This process facilitates the implementation of applications such as motor fault condition classification, fault prediction based on past data, and performance optimisation, among others, by leveraging AI. The analysis of significant changes in these signals is achieved through the utilisation of feature extraction methods. In this study, features were extracted from the database of vibration signals using time-domain statistical methods, frequency-domain analysis, and time–frequency-domain analysis. Initially, the signals were analysed in the time domain, yielding statistical calculations of the mean, RMS, standard deviation, skewness, and kurtosis. The subsequent inspection focused on the effect of the DC offset (average) value of the signals on the evaluation. It was observed that the DC offset did not affect the decomposition. Therefore, the signals were then passed through a 10th-order Butterworth IIR high-pass filter with a cut-off frequency of 5 Hz prior to their use in feature extraction analyses. Subsequent stages of the analysis were then performed on the filtered and pre-processed signals. The frequency-domain feature extraction analyses were conducted using the median frequency, FFT, and WP methods, while the time–frequency analyses were conducted using WPT, EMD, and HHT. Linear Discriminant Analysis (LDA) has been selected as the most fundamental machine learning (ML) algorithm for validating the study’s applicability. The block diagram in Figure 3 shows an overview of the study.

### 2.3. Preprocessing

To speed up analysis and minimise computational load, the compound values were calculated by summing the effectiveness of accelerometer signals in 3 directions (X, Y, and Z) obtained from the database, and the study was carried out by evaluating a single compound signal [43,44].

**Figure 4:**
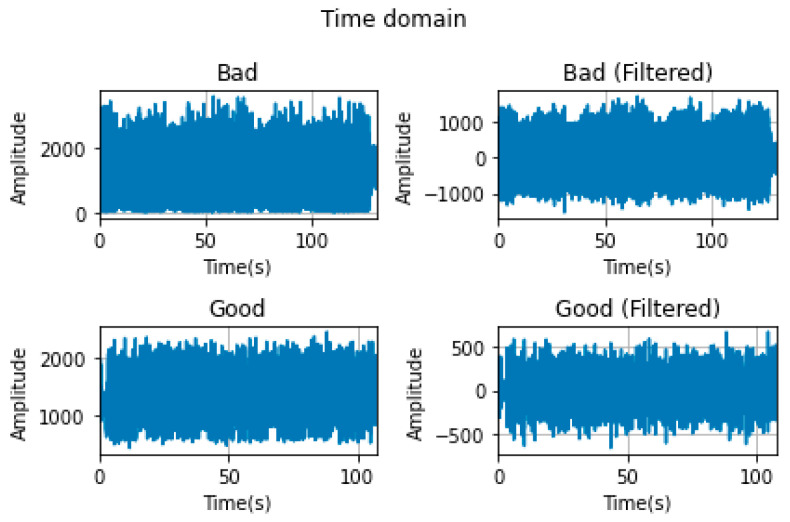
Raw and filtered GP and BP data records.

As illustrated in Figure 4, the filtered and unfiltered images of the sample vibration signals recorded during BP and GP are shown. As shown in the figure, the DC component values of both signals are high. A high DC component may result in complications, including variations in the RMS value during time- and frequency-domain analyses of the signal, a shift in the median frequency towards the DC component, and the suppression of the frequency region of interest. Therefore, it is essential to set the DC components of the signals to zero to eliminate the problems caused by these components. Furthermore, a 10th-order Butterworth IIR high-pass filter with a cut-off frequency of 5 Hz was designed in the preprocessing stage prior to feature extraction, and the signals were passed through it.

Figure 4 clearly shows that the average value of both raw signals is high. This value causes problems with spectral measurements.

### 2.4. Time-Domain Analysis (TDA)

Features are extracted from the time domain by applying various mathematical formulations to the time-axis information, thereby facilitating the retrieval of the underlying signal information. The theoretical information of the time-series analyses evaluated in this study is given below. In the mathematical expressions used in the time-domain calculations that follow, ‘X’ denotes the sample sequence obtained after digitising the signal, ‘X(i)’ denotes the amplitude of the ith sample, and ‘N’ denotes the total number of samples in the sequence.

The *mean* value calculation represents the average of the values obtained from the sampled signals. The calculation of the mean value of any signal is performed by the equation given in Equation (1) [21,45,46,47,48].

(1) $$Mean\;\left(µ\right)=\frac{1}{N}\sum_{i=1}^{N}X\left(i\right)$$

The *RMS* value is employed to describe the power or energy content of signals. This calculation is employed to ascertain its effective value, which is determined as the square root of the sum of the squares of the discrete signal values [21,45,46,47,48]. The RMS value is given in Equation (2).

(2) $$RMS=\sqrt{\frac{1}{N}\sum_{i=1}^{N}{\left|X\left(i\right)\right|}^{2}}$$

The standard deviation of a signal provides numerical information about the distribution of the signal’s data relative to its mean. Equation (3) is the equation used to calculate the standard deviation [21,45,46].

(3) $$Std=\sqrt{\frac{1}{N-1}\sum_{i=1}^{N}{\left(X\left(i\right)-µ\right)}^{2}}$$

Skewness is a statistical indicator of how far the time-series data values deviate from the mean value and the extent to which the data is asymmetric. It refers to the third statistical moment about the mean. Skewness is calculated by the following Equation (4) [21,45,46,47,48].

(4) $$Skw=\frac{\frac{1}{N}\sum_{i=1}^{N}{\left(X\left(i\right)-µ\right)}^{3}}{{Std}^{3}}$$

Kurtosis is a statistical property used to evaluate the peak shape of a signal, indicating whether it is relatively flat or sharply peaked. In summary, the parameter quantifies the extent to which the peak signal intensity deviates from the standard value. Kurtosis is given in Equation (5) [21,45,46,47,48].

(5) $$Krt=\frac{\frac{1}{N}\sum_{i=1}^{N}{\left(X\left(i\right)-µ\right)}^{4}}{{Std}^{4}}$$

In this context, “*µ*” denotes the average of the signal, and *Std* represents standard deviation.

The *median* value of a time-domain dataset *x*_1_, *x*_2_, …, *x*_*n*_, sorted in ascending order as *x*_(1)_ ≤ *x*_(2)_ ≤ ⋯ ≤ *x*_(_*_n_*_)_, is given by:

(6) $$Median=\;\left\{\begin{array}{c} x_{\left(\frac{n+1}{2}\right)},\;\;\;\;\;\;\;\;if\;n\;is\;odd\\ \frac{x_{\left(\frac{n}{2}\right)}+x_{\left(\frac{n}{2}+1\right)}}{2},if\;n\;is\;even \end{array}\right.$$

The median value is particularly useful in time-domain analysis for datasets with outliers or non-Gaussian distributions, as it is less sensitive to extreme values than the mean.

### 2.5. Frequency-Domain Analysis (FDA)

Given the repetitive and oscillatory nature of rotating mechanical systems, it is crucial to extract frequency components from signals for subsequent utilisation in classification and regression analysis algorithms. The evaluation of these features is undertaken to ascertain the frequencies at which anomalies in the functioning of signal-generating systems are predominant. In this study, the efficacy of MF, FFT, and WP for extracting frequency features was evaluated.

#### 2.5.1. Median Frequency (MF)

MF is a feature used to evaluate PSD. The focus of this feature is on half of the energy distribution [49]. It is crucial for understanding the signal’s symmetry or the spectral density’s equilibrium. For a discrete PSD, let *S*(*f_i_*) be the PSD at discrete frequencies *f_i_*, where *i* = 1, 2, …, *N* and Δ*f* is the frequency resolution. The total power is approximated as:

(7) $$P_{Total}\;\approx \;\sum_{i=1}^{N}{S(f}_{i})∆f$$

The median frequency *f_med_* is the frequency *f_k_* such that:

(8) $$\sum_{i=1}^{k}{S(f}_{i})∆f\approx \;\frac{1}{2}P_{Total}$$

discrete frequencies are finally interpolated to determine the exact *f_med_*.

#### 2.5.2. Fast Fourier Transform (FFT)

This process allows a signal to be separated into its different frequency components. The application of the Fourier transform in real sequences is challenging due to the computational requirements. Overcoming this challenge, the Discrete Fourier Transform (DFT) is a discrete version of the Fourier transform for digital signals. The FFT algorithm has been developed as a sophisticated computational tool that enables the expedited analysis of voluminous datasets. This is achieved by the algorithmic simplification of the computational requirements of DFT. Equation (9) presents the mathematical expression of DFT [50,51,52].

(9) $$X\left(k\right)=\sum_{i=0}^{N-1}{X\left(i\right)e}^{-j\left(\frac{2\pi ki}{N}\right)},\;k=0,\;1,\;2\ldots ,\;\left(N-1\right)$$

where *X*(*k*) denotes the discrete signal in the frequency domain, *X*(*i*) signifies the discrete signal in the time domain, and *N* represents the number of samples.

In the present study, the vibration signals were subjected to spectral analysis over 0 to 1 kHz at 1024 points after application of a 5 Hz high-pass filter.

#### 2.5.3. Welch Periodogram (WP)

The Periodogram is a fundamental mathematical technique used to determine the PSD. The WP method was developed to overcome the shortcomings of the Periodogram, including high variance and inadequate resolution. This method provides a stable PSD with lower variance. In this method, the signal is initially divided into segments, and subsequently, each segment is associated with a specific windowing function. The Fourier transform and power spectrum of each segment are calculated, and the power spectra of the segments are centred. WP values $P_{x}\left(f\right)$ are calculated Equation in (10).

(10) $$P_{x}\left(f\right)=\frac{1}{L}\sum_{l=1}^{L}\frac{1}{N_{w}}{\left|\sum_{n=0}^{N_{w}-1}x_{l}\left[n\right]w\left[n\right]e^{-j2\pi fn}\right|}^{2}$$

where *L* refers to the number of segments, *N_w_*, denotes the segment length, *w*[*n*], signifies the windowing function, and *x_l_*[*n*], represents the signal sample in the *l*th segment [53,54,55,56].

### 2.6. Time–Frequency-Domain Analysis (TFA)

Time–frequency-domain methods are used to extract features from time-varying signals, which contain both time and frequency information. In this study, the WPT, EMD and HHT methods are preferred for time–frequency-domain feature extraction.

#### 2.6.1. Wavelet Packet Transform (WPT)

WPT has been proven to be a powerful and adaptive method for time–frequency feature extraction in the analysis of non-periodic signals with instantaneous variations, such as vibration signals. To analyse the signals in more detail, it is possible to filter both high- and low-frequency components at different scales [57,58,59]. It is possible to define WPT as an extension of DWT. The method under consideration involves decomposing the signal’s sub-components into smaller sub-layers. Equations (11) and (12) present the equations for low-pass and high-pass filters of the wavelet function for WPT [58,59].

(11) $$w_{2j}=\sqrt{2}\sum_{k}h(k)w_{j}(2t-k)$$

(12) $$w_{2j+1}=\sqrt{2}\sum_{k}g(k)w_{j}(2t-k)$$

where *k* is the filter coefficient, *w2j* is the high-pass filter, *w2j*+1 is the low-pass filter, *h*(*k*) is the low-pass filter coefficient, and *g*(*k*) is the high-pass filter coefficient. The wavelet packet node energy is given by Equation (13).

(13) $$E_{j,n}=\sum_{k}w_{j,n,k}^{2}$$

where *j* represents the scaling parameter, *k* represents the translation parameter, and *n* represents the oscillation parameter [60,61].

#### 2.6.2. Empirical Mode Decomposition (EMD)

The distinguishing features of this method, which is frequently applied to both linear and nonlinear signals, are its directness and adaptability. The fundamental functions used in the data processing stages are derived directly from the signal, thereby providing greater adaptability. The effectiveness of the system in analysing nonlinear vibration signals is well recognised.

The EMD decomposes oscillatory components, known as IMFs, by shifting. In this method, all local minimum–maximum points of the signal *X*(*t*) are determined. The next step involves clustering local maximum points to form the upper envelope and local minimum points to form the lower envelope. In Equation (13), *m*_1_ denotes the average of all envelopes. The process of shifting culminates when all envelopes are found to be in a state of symmetry. Equation (14), which shows the IMF calculation, is given below [62,63].

(14) $${IMF}_{1}=x\left(t\right)-m_{1}$$

In the next decomposition stage, *IMF*_1_ serves as the new input data for the proto-IMF. In this case, *c*_1*k*_ is the kth IMF component, which can be found using Equation (15).

(15) $$c_{1k}={IMF}_{1(k-1)}-m_{1k}$$

The symmetry condition, denoted by *k*, can be achieved after the elimination process.

#### 2.6.3. Hilbert–Huang Transform (HHT)

This methodology is considered a highly effective technique for analysing signals generated by complex, nonlinear systems. The methodology described herein employs the EMD and the Hilbert transform as functions. This methodology involves decomposing the signal using EMD, followed by analysing the resulting IMFs with a Hilbert transform, thereby yielding a more comprehensive time–frequency–energy description [64,65]. The following IMF and Hilbert transform formulations are given in Equations (16) and (17).

(16) $$x\left(t\right)=\sum_{İ=1}^{n}{IMF}_{i}\left(t\right)+r(t)$$

(17) $${\hat{x}}_{\left(t\right)}=\frac{P}{\pi }\int_{-\infty }^{\infty }\frac{x(\tau )}{t-\tau }d\tau$$

The abbreviation ${IMF}_{i}\left(t\right)$ denotes the *i*th IMF component, while *r*(*t*) is the residual signal. The parameter, *P*, is the Cauchy principal value, and *x*(*τ*) is the real signal. The quantity ${\hat{x}}_{\left(t\right)}$ is known as the virtual part of the analytical pair, and it is obtained by means of a process known as Hilbert transformation. Utilising *x*(*t*) and *y*(*t*) in conjunction with Equation (18), the analytical signal *z*(*t*) is derived [63,66].

(18) $$z\left(t\right)=x\left(t\right)+iy\left(t\right)=a(t)e^{i\varphi (t)}$$

The instantaneous frequency for each IMF is given by Equation (19).

(19) $$\omega_{i}(t)=\frac{d\emptyset_{i}(t)}{dt}$$

where $\emptyset_{i}(t)$ denotes the phase obtained by the Hilbert transform for ${IMF}_{i}\left(t\right)$. $\omega_{i}(t)$ is the instantaneous phase for each IMF [63,66].

### 2.7. Linear Discriminant Analysis (LDA)

Linear Discriminant Analysis (LDA) is a powerful supervised learning algorithm used for both classification and dimensionality reduction. Its main purpose is to find a subspace (projection space) that best separates samples belonging to different classes. The fundamental working principle of LDA is to maximise the between-class scatter (*SB*) while minimising the within-class scatter (*SW*). This situation is mathematically expressed by Fisher’s linear discriminant criterion:

(20) $$J\left(\omega \right)=\frac{\omega^{T}S_{B}\omega }{\omega^{T}S_{w}\omega }$$

Here, *ω* represents the optimal vector onto which the data will be projected. LDA follows a systematic mathematical process to maximise the separability between classes in the data. The process begins with calculating the mean vectors (*μ_i_*) of the features for each class in the dataset. Then, the within-class scatter matrix (S_W_), which represents the distribution of classes within themselves, and the between-class scatter matrix (*SB*), which determines the relative positions of the classes, are created. The main objective of the algorithm is to identify the linear components that best separate the classes by finding the eigenvalues and eigenvectors of the $S_{w}^{-1}S_{B}$ matrix. The obtained eigenvectors define the new subspace onto which the data will be projected; the eigenvalues indicate the discriminative power (variance amount) of this subspace. In the final step, the data is projected onto these new dimensions, achieving both dimensionality reduction and optimisation of classification performance.

## 3. Results

As indicated in the study, the methods mentioned are applied to the signals contained in the database, and the results of this application are reported in detail.

### 3.1. Time-Domain Analysis Evaluations

In TDA, mean, RMS, standard deviation, skewness, kurtosis and median values of the filtered signals were calculated. The values obtained were then used to attempt to make distinctions between GP and BP. However, a comparison of the values shows that time-domain analysis is insufficient for discrimination. The comparison of the raw and filtered signals obtained in the time-domain analysis is shown in Table 1.

According to Table 1, when the mean values of the signals are compared, it is seen that the mean values of the unfiltered BP and unfiltered GP signals are not very different from each other. Therefore, it is considered important to filter the DC offset from the signals using a high-pass filter to remove its dominance in time- and frequency-domain analyses. On the other hand, the table shows that the RMS and STD values of unfiltered signals are not discriminative. Since their mean values are zero, the RMS and STD values of the filtered signals are equal, while the BP and GP discriminations come to the fore. Moreover, the skewness value is not discriminative for both filtered and unfiltered signals. On the other hand, the kurtosis difference between the unfiltered and filtered signals is more pronounced. In the unfiltered signals, the kurtosis value is 1.08 in BP and 2.87 in GP. In the filtered signals, the kurtosis value of 1.52 in BP is 5.06 in GP. Therefore, kurtosis can be used as a discriminative parameter. However, when the standard deviations of these values are analysed, it is found that the standard deviation in BP is 2.43 and in GP is 10.33. This value also undermines the reliability of kurtosis, indicating that kurtosis alone is not significant. On the other hand, the median has a standard deviation of 20.44, which reduces its reliability. As shown in the table, TDA is inadequate for feature extraction in fault detection and will be insufficient for the evaluation. In this case, more advanced signal processing techniques are needed.

### 3.2. Frequency-Domain Analysis Evaluations

The inadequacy of the TDA of the vibration signals used for BP and GP decomposition led to the focus on frequency analysis in this study. Therefore, in the present study, FFT analyses were performed in the first stage of the FDA. Afterwards, the same procedures were performed with Welch periodogram, WPT and HHT methods, respectively.

**Figure 5:**
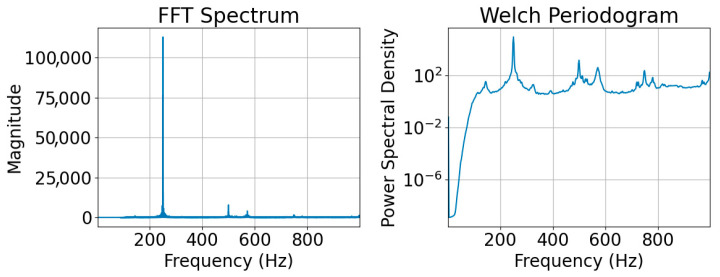
Comparison of FFT and Welch periodogram methods.

After preprocessing, the FFT comparisons of the filtered signals show sharp frequency values and when GP and BP operations are compared, it is seen that separation operations are not possible owing to the limitation of feature extraction. Nonetheless, the application of advanced signal processing methods has enabled the identification of novel features and decomposition patterns within frequency components. Therefore, during the study, the vibration signals were subjected to time–frequency analysis after the FFT process, and the results were compared. Figure 5 showed that a single spike frequency typically stood out after the FFT analysis. However, when the Welch periodogram was analysed, it was evident that other harmonics were also present. Nonetheless, new components, such as median frequency, which can be calculated using the Welch periodogram, can be used to distinguish between GP and BP.

### 3.3. Time–Frequency-Domain Analysis Evaluations

WPT analysis was performed on the vibration signals for BP and GP decomposition as further analysis. Within the scope of WPT analyses, the level and different wavelet types were tested and the results observed. The study’s experimental observations indicate that the most effective WPT types are Daubechies, Symlet, and Coiflet wavelets. However, it has been shown that the performance of these wavelet types is generally similar. Although the performances were close, sym4 was selected as the best wavelet function based on the overall difference compared to the others. A comparison of the wavelet’s frequency resolution at low levels demonstrates its limitations in this regard. Therefore, mathematical entropy optimisation, frequency resolution analyses, statistical security, and sensitivity analyses have been carried out. It is well established in the literature that the WPT technique offers a much finer frequency resolution than standard methods by further resolving detailed information. Frequency resolution: Level 7 provides a bandwidth of 7.81 Hz, thereby ensuring the minimum resolution required to distinguish fault harmonics. Lower levels (e.g., Level 5: 31.25 Hz) are unable to make this distinction. Statistical reliability: At Level 7, there are 226 samples in each sub-band; this number meets the recommended threshold of 200 samples for a reliable estimate of high-order moments. At Level 8 and above, the number of samples is insufficient. Entropy optimisation: Shannon entropy was used in this study. The Shannon entropy analysis indicated that level 7 is the inflexion point. The entropy gain during the transition from level 7 to 8 is only 8.3% of that observed during the transition from level 6 to 7 [67]. Sensitivity analysis: Whilst classification performance reached 96.8% at level 7, the increase at level 8 was only 0.3%, which is statistically insignificant according to the McNemar test (*p* > 0.05). These criteria objectively confirm that level 7 represents the optimal balance point in terms of frequency resolution, statistical reliability, and computational efficiency.

**Figure 6:**
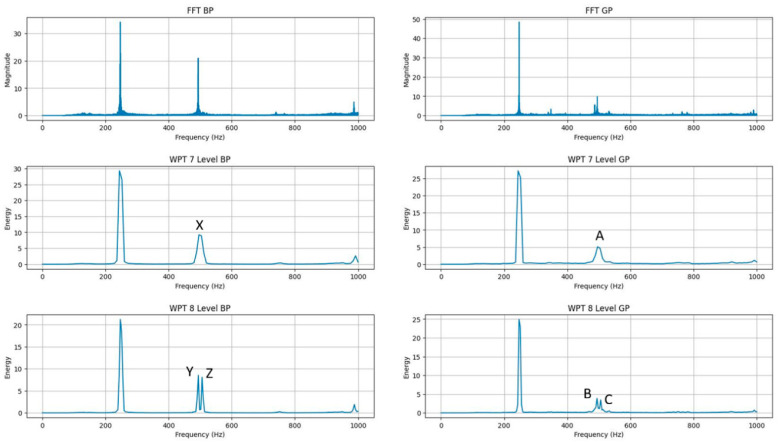
WPT 7 and WPT 8 comparison (185 (GP) vs. 43 (BP)) sym4 wavelet.

Therefore, the presence of distinct frequency bands remains unclear. Consequently, as the level increases, the frequency resolution also improves. As a result, WPT analyses at the 7th level reach the optimal level. Because after the 7th level, the analysis load increases, and the peak frequency value begins to bifurcate. The comparison is shown in Figure 6 (7th level and 8th level comparison).

In Figure 6, the dominant component with a magnitude level of approximately 35–50 units around 250 Hz is observed in the FFT analysis at the top, while a secondary component around 500 Hz is also seen. Meanwhile, within the Wavelet Packet Decomposition (WPT), it is observed that as the level increases, the energy distribution of these components becomes more detailed and the features begin to split. In the analysis of Level 7 WPT, the energy density in the 500 Hz region is observed as a single broad peak (approximately 5–10%) labelled “X” for BP and “A” for GP. When the separation depth is increased to Level 8, these individual structures begin to split, increasing frequency selectivity, while the separation characteristics split as well. In particular, the Y-Z pairs for BP and the B-C pairs for GP, seen in the sub-graphs, are divided and fall within an energy ratio range of approximately 4–8%. Therefore, while the frequency resolution of the system at level 8 shows a characteristic improvement at this level, on the other hand, as expressed in the FFT analysis and WPT level 7 analysis, the discrimination ratio is high; however, at level 8, this ratio decreases (from 5–10% levels to 4–8%).

**Figure 7:**
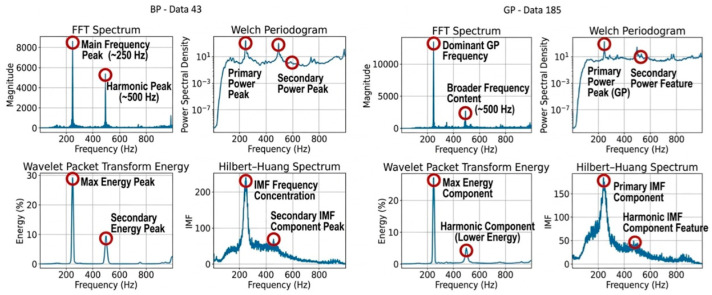
GP and BP comparison of FFT, Welch periodogram, WPT and HHT methods for filtered signals.

On the other hand, HHT analyses, which are the most common method used in the literature for analysing vibration signals, were realised in the study. In contrast to the sharp frequency variations that occur in other methods, HHT analysis can provide more comprehensive inferences about the signal due to the spread around the peak frequency. These changes are expressed in Figure 7.

In Figure 7, spectral analyses, including FFT Spectrum and Welch periodogram analyses, were performed to determine the dynamic characteristics of the system. In both datasets (BP-Data 43 and GP-Data 185), prominent frequency components concentrated around approximately 250 Hz and 500 Hz clearly reveal the system’s periodic operating characteristics and dominant cut-off frequencies. While Welch periodogram and FFT spectra indicate a low noise floor at low frequencies, WPT analysis shows that approximately 25–30% of the total signal energy is concentrated in the fundamental frequency band, and the HHT confirms instantaneous frequency variations and modulation effects through IMF. In particular, the power spectral density obtained using the Welch method provides more reliable energy distribution by removing noise through signal segmentation, thereby offering a stable basis for median frequency (FMED) calculations. The fundamental difference between the BP and GP datasets, reflected in the amplitude variations in the secondary harmonics, indicates that these spectral features can serve as distinctive criteria for monitoring tool wear or changes in processing parameters during CNC operations. However, unlike traditional methods, HHT analysis, which can separate non-stationary signal components, provides critical insights into the physical origins of non-linear interactions within the system through the identified secondary IMF peak points. While the spectral centroid from the Welch spectrum indicates the stability of the system’s overall operating regime, instantaneous frequency changes in the HHT spectrum, along with the determined critical threshold values, enable highly precise characterisation of the system’s features in both the frequency and energy domains.

After a detailed review of the entire study, it was determined that both filtered and unfiltered signals were analysed and features extracted in TDA analyses. Subsequently, as a subset of the FDA, several methods were applied, including FFT, Welch analysis, and median frequency calculations, and the results of these analyses were meticulously compared with a variety of parameters, levels, and wavelet functions. The present study has experimentally demonstrated that the optimal function for WPT is sym4 and that the most effective decomposition level is 7. However, given that the frequency analyses conducted thus far revealed spikes, evaluating solely in the frequency dimension is inadequate. Therefore, the focus is on the advantages of EMD and HHT analyses for enhancing discriminative analyses. In summary, a detailed analysis and investigation of the FFT, Welch, WPT, and HHT methods reveals the advantages of EMD and HHT analysis in terms of the extent of feature extraction.

**Figure 8:**
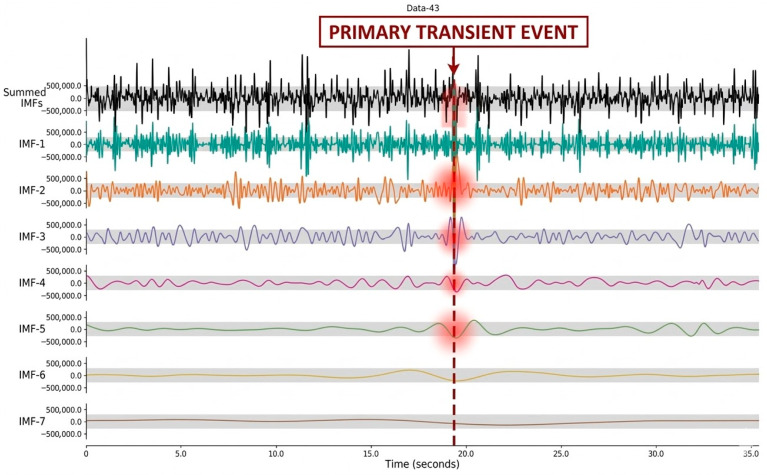
EMD of data in BP class.

The IMF values were extracted via EMD analysis, and the RMS value of each IMF signal was calculated. The values obtained were emphasised as important distinguishing features. The EMD of data evaluated in BP class is shown in Figure 8.

The EMD was performed at seven levels. In Figure 8, the red color blocks represent the primary transient events and energy spikes, the gray shadows indicate the amplitude thresholds for structural vibration monitoring, and the different colors are used to distinguish between individual IMF layers. The “Summed IMFs” graph at the top represents the original signal structure, while the signals from IMF-1 to IMF-7 in the lower layers reflect the hierarchical separation of the data from high frequency to low frequency. The vertically aligned energy concentration occurring at approximately the 17.5 s mark indicates that there is a fundamental change in the temporal character of the process at this point. This temporary anomaly has been isolated as a sudden concentration of energy and a sharp amplitude spike, particularly in the IMF-2 and IMF-3 layers that contain high- and mid-frequency components. When examining the IMF-5 component, which represents the low-frequency dynamics of the process, a more widespread and continuous energy fluctuation is observed at this level, in contrast to the ‘impulse’ characteristic seen in the high-frequency layers. This response at the IMF-5 layer scientifically demonstrates that the resulting anomaly is not limited to a micro-scale crack at the cutting edge, but also affects the system’s overall mechanical impedance and its real-time structural vibration balance. As a result, the sudden transient spikes in high-frequency IMFs and the structural projection in IMF-5 academically confirm that the operation should be classified within the ‘BP’ category from this point onwards, and that the system’s steady-state has been disrupted by a structural impact.

### 3.4. Feature Selections

**Figure 9:**
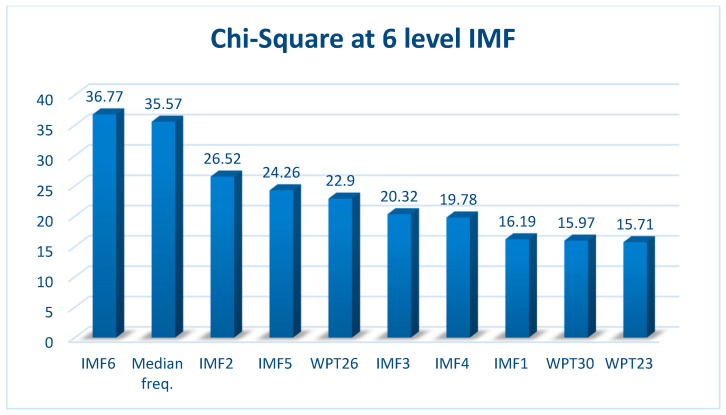
Chi-square feature selection results.

The study compared features extracted from TDA, FDA, and TFA analyses using chi-square tests to quantitatively demonstrate the advantages of each method [68]. The chi-square test for top ten features having the highest scores is shown in Figure 9.

Chi-square test evaluation was extracted for the TDA, FDA and TFA features (there are a total of 289 features while 7th level WPT, and 6th level EMD are performed), and the BP vs. GP discrimination performances were compared. EMD analyses were evaluated at five to ten levels for each characteristic. It is observed that the optimum EMD is level 6. Since median frequency values are identified as the most effective features in decompositions of five levels or fewer. When the 6th level EMD is performed, it is seen that the discrimination of IMF values increases, and the IMF6 value reaches the highest chi-square value. At the 7th level and subsequent decompositions, it is observed that there is a decrease in the chi-square values of IMF values. This plot suggests that IMF6 and median frequency values may exhibit more significant or distinct features in the dataset. In contrast, IMF2, IMF5, and WPT26 show a decline in chi-square values, indicating reduced statistical significance or variability.

Considering all these feature selection evaluations, it is seen that EMD and HHT analyses of the vibration signals provide important features for GP and BP decomposition. The implementation of these features within more comprehensive artificial intelligence methods demonstrates the potential to achieve high-precision BP and GP discrimination during CNC production using large datasets.

**Figure 10:**
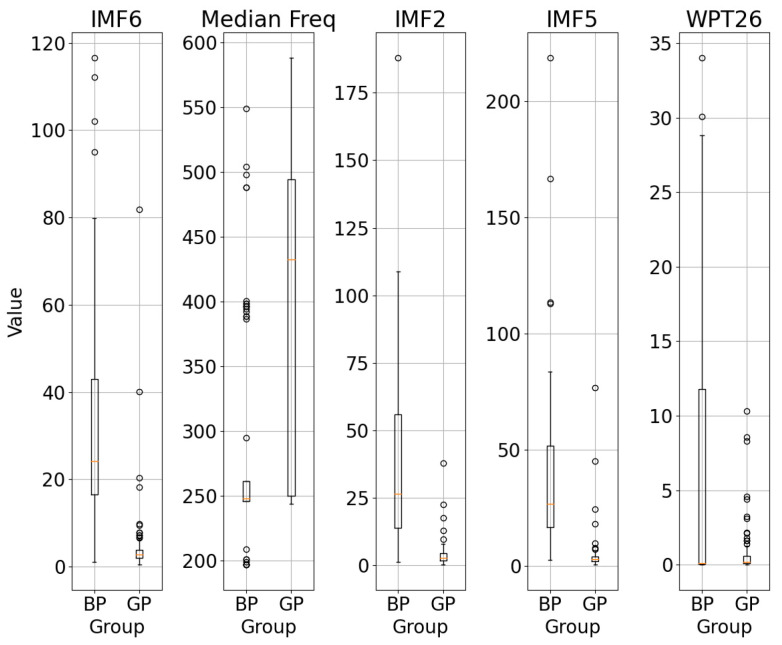
Box plot representation of the five most discriminative parameters.

The box plot representation of the five features with the highest discriminative ability obtained after the chi-square tests is shown in Figure 10.

As shown in Figure 10, the differences between the BP and GP groups were evaluated using box plots, and the discriminative effect of various features was analysed. Feature discrimination was assessed by considering the median difference between groups, the width of the data distributions, and the level of overlap between bins (interquartile range).

Although the chi-square test scores for IMF2, IMF6, and median frequency are high, the IMF5 feature can stand out with some advantages over the others. The evaluation revealed that the IMF5 feature showed the greatest differences between the BP and GP groups. In the BP group, IMF5 values are represented by a high median and a wide distribution, whereas in the GP group these values are concentrated at rather low levels. The observation that the boxes between the two groups do not overlap demonstrates that IMF5 acts as a robust discriminator.

Nevertheless, the IMF2 feature shows comparable levels of discriminative power. While IMF2 values were elevated in the BP group, the GP group showed a low, narrow distribution. This finding implies that IMF2 could effectively discriminate between groups.

Although the WPT26 and median frequency features show a certain difference between the groups, their discriminatory power is lower compared to IMF2, IMF5 and IMF6 due to greater overlap between the boxes. Furthermore, additional feature extraction methods such as Fourier analysis and WPT are required for the WPT26 and median frequency features.

LDA has been selected as the most fundamental machine learning (ML) algorithm for validating the study’s applicability. LDA clearly demonstrates the impact of feature performance on ML algorithms in this study, as it does not require parameter selection for both feature selection and classification problems. Features selected using the chi-square method were sequentially applied to the LDA inputs in the form of binary, ternary, quaternary, and quinary combinations. The entries were subjected to 5-fold cross-validation (5-FCV) using LDA throughout the study. The accuracy values and standard deviations for the top 20 combinations are shown in Table 2.

When Table 2 is evaluated, it clearly demonstrates how the model’s performance is optimised as the number of features increases. Accordingly, Table 2, the combinations “FMED + IMF2 + IMF5 + WPT26”, “FMED + IMF2 + WPT 26 + WPT30”, and “FMED + IMF2 + IMF5 + WPT26 + WPT30” emerge as the most successful combinations with an accuracy value of 91.48%. Furthermore, the fact that the score does not change when switching from a 4-feature to a 5-feature combination indicates that the model has reached saturation with this feature set. In signal processing-based classification models, optimising the feature space is critical both for reducing computational costs and for enhancing the model’s generalisation ability. Therefore, the results from all combinations, the maximum accuracy rates, and the performance evaluations are presented in Table 3.

According to Table 3, the single- and double-feature sets (IMF6, FMED) achieved a basic level of discrimination, with an accuracy of 90.08%. However, upon transitioning to a triple combination (IMF6, FMED, IMF5), which more accurately reflects the signal’s characteristics in both the time and frequency domains, the accuracy rate increased to 90.79%. The highest level of success has been achieved with the integration of four features (FMED, IMF2, IMF5, WPT26) at a rate of 91.48%. This situation demonstrated the spectral dominance of the FMED feature, while the time–frequency components based on IMFs and WPTs showed their discriminative power at the micro level. Based on the experimental analyses, it was determined that FMED, which was present across all high-performance combinations, had the highest discriminative power. The feature importance order was provided by IMF2, IMF5, and WPT26, respectively, offering complementary information. The maximum accuracy rate of 90.08% achieved in single and binary combinations has been improved with the feature fusion strategy. By including the IMF2, IMF5, and WPT26 features that carry the time–frequency-domain information into the system, the model’s prediction accuracy has been increased to 91.48%. The fact that the accuracy rate remains constant in combinations containing five or more features indicates that the quadruple set of FMED, IMF2, IMF5, and WPT26 is the ‘optimal feature subset’ for the dataset and that the model has reached information saturation at this point. On the other hand, although the main feature that provides the highest success rate at 90.08% in binary combinations is the IMF6, in triple and quadruple combinations, this gives way to the IMF2 and IMF5 features, which offer a broader frequency characteristic, and due to these new sets reaching higher accuracy rates such as 91.48%, it is outside the ‘optimal’ ranking.

Consequently, FMED, IMF2, IMF5 and WPT26 were identified as the most effective features for distinguishing between BP and GP groups. These features can be prioritised in further analysis or classification algorithms.

## 4. Discussion and Conclusions

This study investigated the effectiveness of feature-extraction methods based on vibration signals for the automatic discrimination of GP and BP during CNC milling. Long-term 3-axis vibration data collected in the real-time production environment were evaluated using various signal processing and analysis methods.

In the first stage, basic statistical properties such as mean, RMS, standard deviation, skewness, kurtosis, and median were analysed using time-domain methods; however, it was found that these methods alone were insufficient for BP and GP discrimination. Although some parameters, such as kurtosis and median, can be discriminative, especially in analyses of filtered signals, their reliability is limited.

In the frequency-domain analyses, the spectral structure of the signals was examined using the FFT, median frequency, and Welch methods. After these analyses, the WPT and HHT methods, which can provide more detailed information in the time–frequency domain, were applied. While the sym4 wavelet and 7th-level decomposition yielded the most successful results in WPT analyses, HHT analyses increased discrimination power by revealing the propagation of frequency components.

In the final stage of the analysis, the IMF components obtained by the EMD method were statistically evaluated using a chi-square test. The evaluation yielded findings indicating that the FMED, IMF2, IMF5, and WPT26 components were the most robust features for BP and GP discrimination, respectively. The box plots revealed that these features clearly distinguished the groups.

Considering that the minimum sample size N in the data and the sampling frequency of 2 kHz are taken into account, the EMD can theoretically be decomposed into up to 14 levels (log_2_N as the theoretical upper limit) [69,70]. However, in practice, when criteria such as the correlation coefficient, energy contribution ratio, and statistical significance test are considered, it has been suggested that the IMF range corresponding to the mid-frequency band, between 5 and 10, is appropriate [71]. The determination of the EMD level in the study is based on a systematic experimental evaluation process carried out within the range of levels 5 to 10, rather than a random selection. In the statistical analyses conducted, it was observed that at level 5 and below, the discriminative power of IMF components was low due to the broad frequency range of each IMF component, and other features such as median frequency were more prominent at these levels. However, when the separation depth was increased to level 6, the statistical accuracy of the IMF components significantly improved, and in particular, the IMF6 component achieved the highest chi-square (ki-kare) score at this level, demonstrating the strongest discrimination between GP and BP classes. When the analyses are extended to level 7 and above, a decrease in the chi-square scores of the IMF values has been observed due to the narrowing and splitting of the frequency bands of each IMF value. This situation has shown that deeper disassemblies decrease statistical significance rather than increase the distinctive information in the dataset. Therefore, level 6 has been identified as the optimal separation depth in terms of both computational load and feature selection success; this is also confirmed by box plot analyses, which show that the most effective parameters, such as IMF2, IMF5, and IMF6, provide the clearest separation at this level.

In the literature, the number of studies using the same dataset and using the feature extraction methods in the current study is limited. In a study, Nayana et al. [46] focused on bearing-fault diagnosis in induction motors, using only time-domain statistical features, such as waveform length, slope sign changes, and zero crossings. The study relies on the Case Western Reserve University bearing dataset and applies classifiers such as SVM, LDA, and Naive Bayes. The key goal of this work is to achieve high classification accuracy with low computational complexity by using a small set of effective statistical features, avoiding the complexity of TFA. The study by Altaf et al. [21], one of the most closely related to the present study, aims to achieve high-accuracy classification using a lower-dimensional, statistically based approach. On the other hand, Çekik et al. [72] do not explore multiple signal processing domains. Instead, they focus on machine learning classification of vibration data from old (brownfield) CNC machines using various versions of Extreme Learning Machines (ELMs), including MELM, CSELM, and SELM. The primary aim is not to compare signal features, but to compare the performance of ELM-based classifiers on real-world data collected over two years using Bosch CISS sensors. Although frequency analysis is mentioned (e.g., spindle speed harmonics), there is no in-depth analysis of TDA, FDA, or TFA methods, nor any use of EMD/HHT. Ali, J. B. et al. [37] focus on REB fault diagnosis using EMD and artificial neural networks, leveraging run-to-failure vibration data from the Intelligent Maintenance Systems (IMS) dataset to classify seven bearing states (healthy, degraded, and failure states for roller, inner race, and outer race). It emphasises EMD energy entropy, statistical features, and the J criterion for feature selection, without mentioning CNC milling, triaxial accelerometers, or the timeframe 2018–2021. The emphasis is placed on faults that are specific to bearings and the classification of artificial neural networks. On the other hand, Toma et al. focus on classifying bearing faults in REB within induction motors, using a dataset from a custom testbed at the University of Ulsan. It employs EEMD to decompose vibration signals, selects IMFs based on kurtosis, reconstructs the signal, and uses Continuous Wavelet Transform to generate 2-D spectrogram images for a Convolutional Neural Network to classify four bearing conditions (normal, inner, outer, and roller faults), achieving 99.19% accuracy [73]. The present study, by comparison, conducts a thorough evaluation of the TDA and FDA analyses, demonstrating that HHT and EMD analyses are more effective in capturing the multifaceted nature of vibration signals. In this context, the present study demonstrates the efficacy of more sophisticated and advanced time–frequency analyses and provides a more efficient approach to BP and GP discrimination. In summary, when similar studies in the literature are compared with the present study, it is seen that studies that use similar methods and have high accuracy are generally used for rotating machine fault detection. On the other hand, the number of studies and datasets related to the classification of defective production during CNC production is limited. The present study focuses on feature extraction for the identification of defective production in CNC manufacturing, with the objective of overcoming this limitation.

The most significant novelty obtained within the scope of the study has been the extraction of the characteristics required for the BP and GP distinction. Studies in the literature have generally avoided complex methods, and no in-depth analysis or research has been conducted on feature extraction and feature selection. At this point, a study focusing solely on feature extraction and selection is planned. It has been demonstrated that the characteristics obtained within the scope of the study will contribute significantly to machine learning and prediction studies. Within the work, it was clearly stated why the HHT and EMD methods were preferred, particularly in the context of Figure 7. Subsequently, in the feature selection section, the discriminative power of the features obtained from these methods was demonstrated using the chi-square test. Furthermore, the identification of prominent features in the BP and GP distinction, which is of great importance in terms of process control in CNC Milling operations, has been addressed for the first time in the literature. This also highlights the study’s originality.

In light of these findings, time–frequency-based features extracted from vibration signals, especially when supported by EMD and HHT analyses, provide a strong basis for early and automatic detection of manufacturing defects in CNC machines. In the future, by combining these analyses with larger datasets and advanced machine learning and deep learning models, it can be aimed to improve production quality and reduce scrap rates.

## Figures and Tables

**Table 1:** TDA comparison table.

Title ^1^	Mean	RMS	STD	Skewness	Kurtosis	Median
MBNF	1150.98	1197.79	321.57	0.6	1.08	1121.91
SBNF	49.53	72.93	96.96	0.35	1.17	39.55
MGNF	1104.86	1132.97	247.61	0.97	2.87	1061.24
SGNF	21.46	29.1	44.32	0.41	2.06	25.8
MBF	0	231.08	231.08	0.04	1.52	3.39
SBF	0.01	104.62	104.62	0.25	2.43	20.44
MGF	0	133.52	133.52	0.06	5.06	0.32
SGF	0	49.45	49.45	0.26	10.33	7.8

^1^ M: Mean of Analysis, S: Standard Deviation of Analysis, G: GP, B: BP, NF: Nonfiltered Signals, F: Filtered Signals.

**Table 2:** LDA accuracy and standard deviation results.

		Features ^1^			Accuracy	Standard Deviation
F1	F2	F3	F4	F5		
FMED	IMF2	IMF5	WPT26		0.9148	0.0307
FMED	IMF2	WPT26	WPT30		0.9148	0.0307
FMED	IMF2	IMF5	WPT26	WPT30	0.9148	0.0239
IMF6	FMED	IMF2	WPT26	IMF3	0.9113	0.0159
FMED	IMF2	WPT30	WPT23		0.9113	0.0253
FMED	IMF2	IMF5	WPT26	WPT23	0.9113	0.0253
FMED	IMF2	WPT26	WPT30	WPT23	0.9113	0.0253
IMF6	FMED	IMF2	IMF5	WPT26	0.9112	0.0199
FMED	IMF2	IMF5	WPT26	IMF4	0.9112	0.0277
IMF6	FMED	IMF2	WPT26	WPT30	0.9112	0.0229
IMF6	FMED	IMF5			0.9080	0.0358
IMF6	FMED	IMF1			0.9078	0.0306
FMED	IMF2	WPT26	WPT23		0.9078	0.0208
IMF6	FMED	IMF2	IMF3	WPT23	0.9078	0.0134
FMED	IMF2	WPT26			0.9077	0.0263
FMED	IMF2	WPT23			0.9077	0.0263
FMED	IMF2	IMF5	WPT23		0.9077	0.0263
IMF6	FMED	IMF2	IMF5	WPT23	0.9077	0.0209
IMF6	FMED	IMF2	WPT26	IMF4	0.9077	0.0179
IMF6	FMED	IMF2	WPT26	WPT23	0.9077	0.0238

^1^ F1, F2, F3, F4, F5: Features.

**Table 3:** Maximum accuracy rates and performance reviews for all combinations.

Feature Combinations	Combination	Max Accuracy %	Computational Cost	Benefit–Cost Ratio	Status
2-feature	IMF6, FMED	90.08	Very Low	High	Fast but Inadequate
3-feature	IMF6, FMED, IMF5	90.80	Low/Medium	Very High	Good Alternative
4-feature	FMED, IMF2, IMF5, WPT26	**91.48**	Acceptable	Maximum	Optimum
5-feature	FMED, IMF2, IMF5, WPT26, WPT30	91.48	High	Low	Redundant

## Data Availability

The datasets analysed during the current study are available in the CNC Machining Data repository; https://www.kaggle.com/datasets/maximilianfellhuber/cnc-machining-data (accessed on 10 February 2026).

## Abbreviations

The following abbreviations are used in this manuscript:

BP	Bad Processing
CNC	Computerised Numerical Control
DFT	Discrete Fourier Transform
DWT	Discrete Wavelet Transform
EEMD	Ensemble Empirical Mode Decomposition
ELM	Extreme Learning Machines
EMD	Empirical Mode Decomposition
FDA	Frequency-Domain Analysis
FFT	Fast Fourier Transform
GP	Good Processing
HHT	Hilbert–Huang Transform
IMF	Intrinsic Mode Function
IMS	Intelligent Maintenance Systems
MEMS	Microelectromechanical systems
MF	Median Frequency
OP	Different operational segments
PSD	Power Spectral Density
REB	Rolling element bearings
SSA	Singular Spectrum Analysis
STFT	Short-Time Fourier Transform
TDA	Time-Domain Analysis
TFA	Time–Frequency-Domain Analysis
WP	Welch periodogram
WPD	Wavelet Packet Decomposition
WPT	Wavelet Packet Transform
